# A next-generation sequencing method for overcoming the multiple gene copy problem in polyploid phylogenetics, applied to *Poa *grasses

**DOI:** 10.1186/1741-7007-9-19

**Published:** 2011-03-23

**Authors:** Philippa C Griffin, Charles Robin, Ary A Hoffmann

**Affiliations:** 1 Department of Genetics, University of Melbourne, Parkville 3010, Victoria, Australia; 2 Department of Zoology, University of Melbourne, Parkville 3010, Victoria, Australia

## Abstract

**Background:**

Polyploidy is important from a phylogenetic perspective because of its immense past impact on evolution and its potential future impact on diversification, survival and adaptation, especially in plants. Molecular population genetics studies of polyploid organisms have been difficult because of problems in sequencing multiple-copy nuclear genes using Sanger sequencing. This paper describes a method for sequencing a barcoded mixture of targeted gene regions using next-generation sequencing methods to overcome these problems.

**Results:**

Using 64 3-bp barcodes, we successfully sequenced three chloroplast and two nuclear gene regions (each of which contained two gene copies with up to two alleles per individual) in a total of 60 individuals across 11 species of Australian *Poa *grasses. This method had high replicability, a low sequencing error rate (after appropriate quality control) and a low rate of missing data. Eighty-eight percent of the 320 gene/individual combinations produced sequence reads, and >80% of individuals produced sufficient reads to detect all four possible nuclear alleles of the homeologous nuclear loci with 95% probability.

We applied this method to a group of sympatric Australian alpine *Poa *species, which we discovered to share an allopolyploid ancestor with a group of American *Poa *species. All markers revealed extensive allele sharing among the Australian species and so we recommend that the current taxonomy be re-examined. We also detected hypermutation in the *trn*H-*psb*A marker, suggesting it should not be used as a land plant barcode region. Some markers indicated differentiation between Tasmanian and mainland samples. Significant positive spatial genetic structure was detected at <100 km with chloroplast but not nuclear markers, which may be a result of restricted seed flow and long-distance pollen flow in this wind-pollinated group.

**Conclusions:**

Our results demonstrate that 454 sequencing of barcoded amplicon mixtures can be used to reliably sample all alleles of homeologous loci in polyploid species and successfully investigate phylogenetic relationships among species, as well as to investigate phylogeographic hypotheses. This next-generation sequencing method is more affordable than and at least as reliable as bacterial cloning. It could be applied to any experiment involving sequencing of amplicon mixtures.

## Background

Polyploid species are numerous and economically important. Cycles of polyploidization and diploidization have recurred throughout the evolutionary history of eukaryotes [1,2], such that many eukaryotic species possess more than the two chromosome sets expected in a diploid. There are polyploid animals, fungi and protists [3,4], but polyploidization has been especially prevalent in flowering plants. The entire angiosperm lineage underwent at least one round of ancient polyploidization early in its evolution [2]. Many family- or lineage-specific polyploidization events have occurred since then (examples reviewed in [5]), and most of our main food and economic crop species are recent polyploids, including cotton, bread wheat, maize, potatoes, brassicas, bananas, tobacco and coffee [6,7].

Polyploidization is often associated with genome plasticity. Chromosomal rearrangements, new transposable element activity, DNA mutation, duplicate gene deletion, gene expression and epigenetic changes are commonly observed with polyploid formation [2,5,6,8]. Polyploidy maintains higher levels of heterozygosity in a population, reducing the occurrence of inbreeding depression, although mutant alleles accumulate in polyploid populations more quickly than in diploids [4]. Polyploids can also adapt faster than diploids as long as beneficial mutant alleles are not masked too strongly by wild-type alleles [4]. Each polyploidization event combines genes in novel ways, potentially producing a lineage with new phenotypes, capable of surviving and adapting to environments outside the range of its parent lineages [5,9,10]. Thus polyploidization may be a vital mechanism for adaptation to rapidly-changing environments, such as those expected under anthropogenic climate change.

Practical difficulties have limited our current understanding of polyploid evolution, diversification and population dynamics. Nuclear DNA sequence is the most informative data source for phylogenetic inference. Haploid organellar sequence data can be useful, but nuclear regions must be included to obtain multiple unlinked markers. These are necessary where the evolutionary history of a lineage is complicated by incomplete lineage sorting or hybridization, because a single marker has a low probability of predicting the true evolutionary tree [11-14]. Nuclear sequence data are often useful in investigating complex evolutionary histories [15-17]. Since polyploids contain multiple distinct copies of each nuclear gene, known as homeologues, it is usually impossible to amplify a homogeneous, single amplicon using PCR. DNA sequencing by the Sanger method can only be performed on a single pure amplicon. If more than one copy is amplified with a particular PCR primer pair, direct sequencing will give double peaks at sites that differ within and between homeologues, and the phase of many such double peaks can not be determined. Furthermore, if there are insertion-deletion polymorphisms (indels) that distinguish between the copies, then direct sequencing will fail because all sites after an indel will be undecipherable double peaks. The resulting practical difficulties with gene sequencing have been well documented [13,16,18].

Several approaches have been used to overcome this problem, but all have drawbacks, in terms of the extra time and cost associated with each method compared to similar work in diploid or haploid taxa. Many researchers have used bacterial cloning to separate gene copies [17,19,20]. This is time-consuming and expensive, meaning that few individuals and few genes are investigated. A single species is often represented by a single individual [17,20,21], which may be adequate for investigating the hybrid origins of species [20], but not for investigating intraspecific variation. Comparisons based on individuals may also lead to the wrong conclusion about the evolutionary history of related taxa, due to undetected incomplete lineage sorting and/or reticulate evolution [11]. Other researchers have designed primers specific to each known homeologue of a multi-copy nuclear gene [16,22], but this method requires extensive preliminary experimentation in poorly-characterized species [13,22,23]. It may also be unsuitable for autopolyploids or some allopolyploid genes for which good distinguishing primers cannot be designed. In some studies, DNA is extracted from a mixture of individuals from a single population [24], but this might not accurately estimate the frequency of alleles in a population, as preferential PCR amplification may occur.

Here we describe a new method that uses the capacity of next-generation sequencing technologies to sequence mixtures of DNA rather than pure PCR products. We sequence multiple gene copies, multiple genes, and multiple individuals in a single run, using barcoded samples. Our method demonstrates the utility of 454 sequencing for phylogenetic inference in a group of polyploid grasses that seem to have evolved under a recent, rapid radiation. It could be applied to any experimental design that aims to sequence amplicon mixtures from many independent samples.

### Study species

Tussock grasses in the genus *Poa *(Poaceae) dominate the alpine communities of the Australian Alps. These grasses were initially lumped as *Poa australis *R. Br. or *P. caespitosa *Forst. f., despite variation in morphology and habitat associations [25]. More recently, approximately 50 Australian *Poa *taxa have been described [25], about 12 of which occur in the alpine and/or subalpine region [25,26]. The genus *Poa *has long been regarded as taxonomically difficult [21,27,28] and even viewed as a massive polyploid complex [29]. The taxonomy of the Australian alpine species is also considered to be imperfect [25,26].

Initial results from a pilot study using microsatellite markers, chloroplast non-coding DNA and nuclear regions amplified in 25 herbarium specimens indicated that these *Poa *species are tetraploid. This was consistent with evidence from a previous study by Patterson *et al. *[21] that found two copies of each of the two nuclear genes analyzed in a range of *Poa *species, and with the very common occurrence of polyploidy in the entire grass family [9].

## Results

### Sequencing results

**Figure 1:**
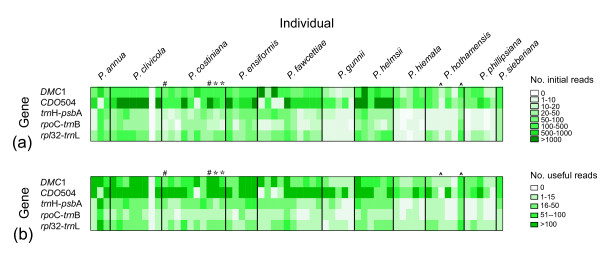
**Number of sequence reads obtained for each marker/individual combination**. **A - **After quality control and barcode deconvolution. Each column represents an individual, grouped by species. *, # and ^ symbols indicate pairs of repeated individuals. **B - **Useful sequence reads remaining after alignment and editing. Note different grey scale. Each column represents an individual, grouped by species. *, # and ^ symbols indicate pairs of repeated individuals.

We submitted a mixture of three chloroplast and two nuclear gene regions amplified in 61 *Poa *specimens from 11 species for 454 sequencing. The ¼ plate 454 sequencing reaction produced approximately 121,000 sequence reads. Read length ranged from 40 to 775 bp (mean ± 1 s.d. = 278 ± 153 bp). Approximately 111,200 (92%) of these reads were successfully matched to one of the gene region primer sequences (Figure 1A). After barcode deconvolution and quality control 70,601 reads remained (Figure 1B).

The barcode success rate was checked using a subset of the *rpl*32-*trn*L reads. Of the 3,448 reads matching at least one of the amplicon primer sequences, 2,732 exhibited the desired amplicon primer + 3-bp barcode + checksum base and so were matched to an individual. A further 656 reads failed to match the amplicon primer + barcode sequence either because they were trimmed at the Adapter A end (through fragmentation after the initial PCR, or through sequencing error) or because of sequencing error(s) in the primer region. Only 60 reads (2.0%) were rejected because of checksum base problems, and these were usually because of a missing checksum base rather than an incorrect base call. All mismatches were discarded during the barcode deconvolution process.

After quality control, sequence error rate was estimated as the percentage of single-base mutations in the *rpl*32-*trn*L region within individuals and was found to be approximately 0.13% (see Methods). Despite efforts to include equal amounts of PCR product for each chloroplast region/individual combination (and four times that amount for each nuclear gene/individual combination), read numbers varied across genes and individuals and showed some species-specific patterns (Figure 1).

### Individual success

Useful sequence (≥1 read per individual) was obtained for 281 out of 320 (88%) of gene/individual combinations (Figure 1B). The resulting sequences are available in GenBank [30] (accession numbers [HQ542308-HQ542469 and HQ594198-HQ594464]). Eight (21%) of the unsuccessful samples dropped out or showed very weak amplification (<4 ng/μl product) at the PCR stage. The rest of the unsuccessful gene/individual combinations appeared to amplify successfully, but failed at the sequencing stage. Most of these involved a chloroplast region and we suspect that they failed because insufficient PCR product was included to ensure useful sequence reads. One sample (a *P. clivicola *individual) produced no sequence reads for any gene. This does not seem to be a barcode-related problem, because the same barcode was successful in another experiment (CR *et al.*, unpublished work). It probably resulted from experimental error (for example, failure to include this individual in the final amplicon mixture).

### Achieving sufficient sequence reads

**Figure 2:**
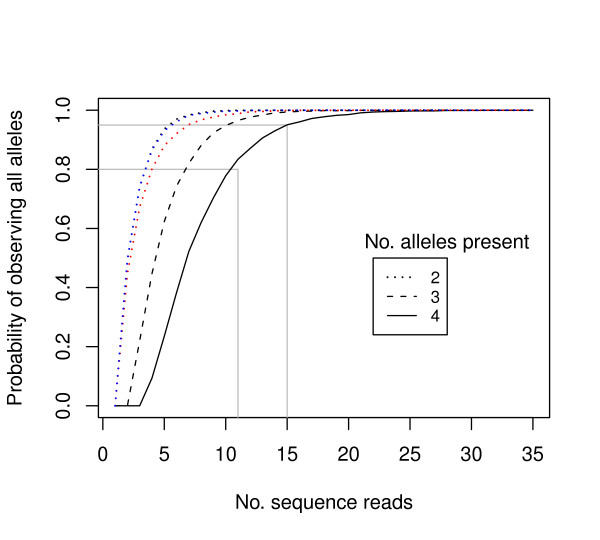
**Probability of observing all alleles for one nuclear region vs. number of sequence reads obtained**. 10,000 draws with replacement were simulated for each sequence read number. Black lines show the case of equal allele proportions where the true allele number is 2 (dotted line), 3 (dashed line) and 4 (solid line). Red and blue dotted lines show the case for the observed copy proportions for CDO504 and *DMC*1 respectively. Grey vertical lines show the read number required to observe all alleles with >0.8 and >0.95 probability when 4 alleles are actually present.

For the chloroplast regions, a single read per individual provided sufficient sequence information, since these regions were single-copy. For the nuclear genes, however, up to four alleles were expected per region (two gene copies with up to two alleles each). Calculating the number of reads required to obtain all existing alleles is an example of the 'Inverse Coupon Collector's Problem' in probability theory [31] and was previously modelled by Joly *et al. *[32]. We simulated the cases of two, three and four existing alleles, assuming that all alleles were amplified evenly, and repeated the simulation 10,000 times for each number of sequence reads (Figure 2). These analyses suggest that at least 15 reads are needed to be 95% sure that all four alleles are sequenced.

We then wanted to check whether the assumption of equal read proportions for each allele was justified. To do this, we regressed the number of copy B sequence reads against the number of copy A sequence reads for each nuclear gene, with the regression forced through the origin (0, 0). A t-test was used to discover whether the regression slope (the B:A ratio) was significantly different from 1 for both nuclear genes. For the CD0504 gene the mean ratio of B copies to A copies was 1.87 ± 0.23 (*t *_55 _= 3.77; *P *< 0.001). For the *DMC*1 gene there were also more B copies with a mean ratio of 1.29 ± 0.11 (*t *_60 _= 2.64; *P *< 0.01). Using these ratios in the simulation algorithm we found that five to seven reads were required to be 95% confident that both copies had been sequenced (Figure 2).

### Repeatability

**Figure 3:**
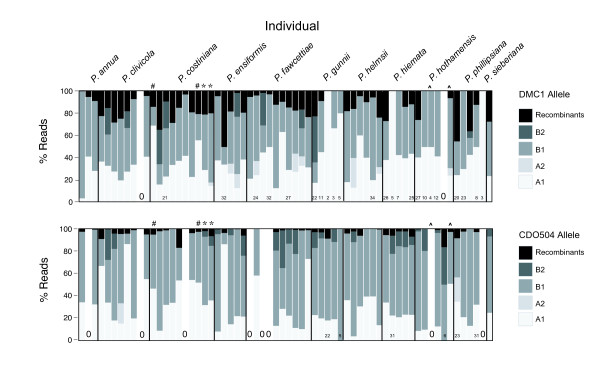
**Percentage of useful reads gained for each nuclear gene copy and allele, including recombinant reads**. The upper panel shows *DMC*1 results, the lower panel shows CDO504. For individual/gene combinations with <50 reads for that gene, the number of useful reads is printed at the base of the column. Within each gene, the gene copies are referred to as 'A' and 'B', and the alleles 'A1' and 'A2', as per the text. *, # and ^ symbols indicate pairs of repeated individuals.

The procedures were repeated (from the DNA extraction stage onwards) for three individuals (two *P. costiniana *and one *P. hothamensis*), with repeats barcoded separately to test the accuracy and repeatability of the entire process. The two repeated *P. costiniana *individuals showed identical alleles at the level of stringency chosen here (≥2 bp difference required to assign different alleles), except for one low-frequency extra *DMC*1 Copy A allele that was found in one individual but not its repeat (Figure 3). On examination, this apparent extra allele probably resulted from a PCR error as it was identical to the other allele except for a unique 2 bp insertion. For the repeated *P. hothamensis *individual, one repeat produced consistently few sequence reads, while the other sequenced well (Figures 1, 3). This was probably due to a low-quality DNA extraction and poor PCR amplification for the first repeat. These two repeats differed by 3 and 2 bp for the *DMC*1 A and B allele respectively, but the alignments from the first individual were unreliable because they were based on only two sequence reads each (Figure 3). In general, the method was repeatable and accurate when a minimum of 2 bp difference was required (out of approximately 400) to assign different alleles.

### PCR Recombination

PCR recombination was judged to have resulted in 302/10,301 (2.9%) of 'useful' reads for CDO504 and 614/4,326 (14%) for *DMC*1. Examining a subset of suspected recombinants in RDP3 [33] supported this judgement (see Methods). At the individual level, recombination frequency (mean ± 1 s.d.) was 3.5 ± 3.6% for CDO504 and 13.6 ± 11.5% for *DMC*1 (Figure 3).

### Polyploidy in nuclear genes

Two distinct copies of each nuclear gene were detected. These were separated by 19 (4.0%) and 35 (8.5%) fixed base differences for *DMC*1 and CDO504 respectively. Furthermore, there were no shared polymorphisms between the A and B copies for either homologue set. The *DMC*1 copies also differed by a 7 bp indel and the CDO504 copies differed by 4 indels 1 to 4 bp in length. No recombination was detected among the two copies for either gene, and the two copies were not in linkage disequilibrium (see Methods). For this reason they were treated as separately-evolving, unlinked loci. *Poa annua *was the expected outgroup because it is native to Europe, though it is now widespread in Australia. Two distinct copies were also detected in this species and a single *P. annua *copy was included in each alignment as an outgroup.

Coding regions of the nuclear genes were either based directly on GenBank matches (*DMC*1) or determined using using Artemis and Artemis Comparison Tool (ACT) software [34,35] on an alignment of corn, rice and sorghum sequences from GenBank (GenBank: 226502914, NC_008396 and 239825523, respectively) and representative *Poa *sequences (CDO504). Coding regions were examined for synonymous and non-synonymous mutations, frameshift mutations or stop codons that would render gene copies non-functional in some individuals. Both *DMC*1 copies had few coding region mutations (DMC1 copy A: one non-synonymous, two synonymous; DMC1 copy B: one non-synonymous, three synonymous). CDO504 copy A exhibited four non-synonymous and six synonymous mutations in the coding region. Overall mutations were rare among individuals for all four gene copies (Tajima's D <0, *P *< 0.05 for coding region and overall sequence).

Initially, CDO504 Copy B was detected to have more than two alleles in most individuals, suggesting an extra gene copy was present. Twenty-four percent of all Copy B reads included a 57-bp deletion in the first intron. This 'short' allele was found in most individuals. These alleles matched Copy B but such a deletion was not detected in any GenBank matches. No secondary structure was detected and so this apparent allele was considered to represent a real gene copy, probably resulting from duplication of CDO504 Copy B. This apparent gene copy was excluded from further analysis but we cannot rule out the possibility that some of the 'long' Copy B alleles may actually be reassorting at this locus.

Within the 'long' Copy B coding region, three non-synonymous and four synonymous mutations were detected at low frequency (Tajima's D < 0, *P *< 0.05 for coding region and overall sequence). Eight individuals had CDO504 copy B alleles with exon insertions that caused frameshift mutations, making the putative protein product non-functional. Since three of these individuals shared a 2-bp insertion, three shared a 5-bp insertion and two shared a 32-base insertion, individual-specific sequencing errors were unlikely and these insertions were considered to be genuine. These frameshift mutations may indicate a loss of function in this gene copy.

**Figure 4:**
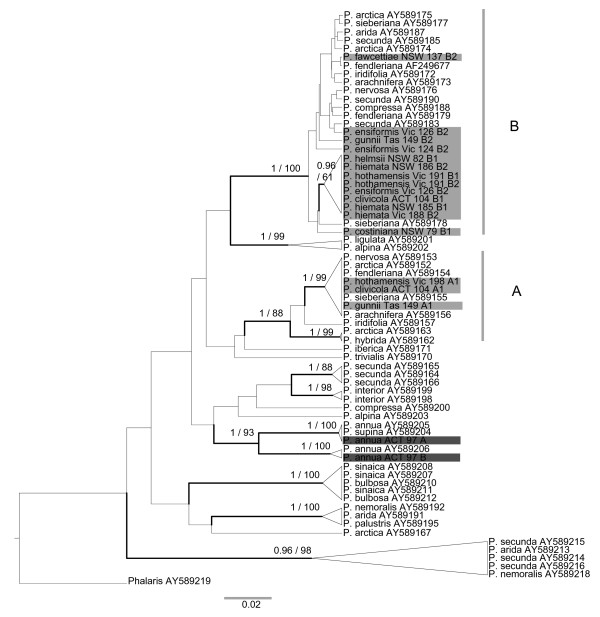
**Phylogenetic tree of *Poa *CDO504 sequences**. Maximum clade credibility tree made using BEAST [91]: 10,000,000 generations, constant population size, GTR+G model with starting substitution matrix and gamma parameter values chosen in ModelTest 3.7. Nodal support values shown are posterior probabilities/maximum parsimony bootstrap percentages (500 replicates). Only nodes with posterior probabilities ≥0.95 or bootstrap percentages ≥80 are shown. Well-supported nodes are also shown with thick lines. Representative sequences from the native Australian species from this study are shaded in grey. Other sequences are from Patterson *et al*. [21], downloaded from GenBank, including *Phalaris *used as an outgroup. All Australian copy A sequences from this study fit clade A (also called A in Patterson *et al. *2005); all Australian copy B sequences fit clade B (called Clade C in Patterson *et al. *2005).

To compare our results to those previously reported for the genus *Poa*, we built a phylogenetic tree using a subset of the Australian CDO504 copy A and B sequences (spanning the diversity observed in each), along with other *Poa *CDO504 sequences obtained from GenBank (see Methods). All of the Australian CDO504 copy A sequences fell within the well-supported sequence class A (as defined by Patterson et al.), and all the CDO504 copy B fell within sequence class C (Figure 4).

### Species differentiation

**Figure 5:**
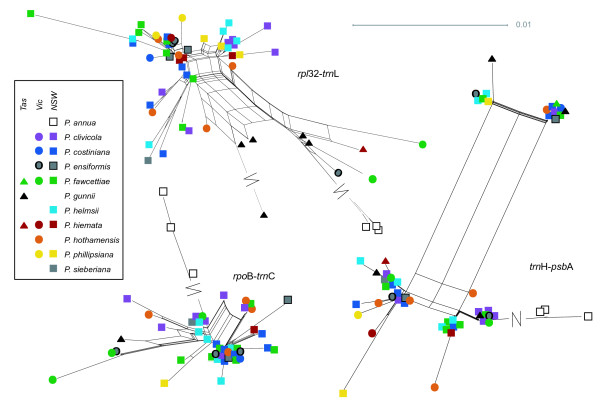
**Networks for the three chloroplast non-coding regions sequenced**. All networks created using the NeighbourNet algorithm in SplitsTree 4.11.3 using the EqualAngle layout option, from distance matrices created in Mega 5 (see text for details). Long splits are indicated by a break and not shown to scale.

**Figure 6:**
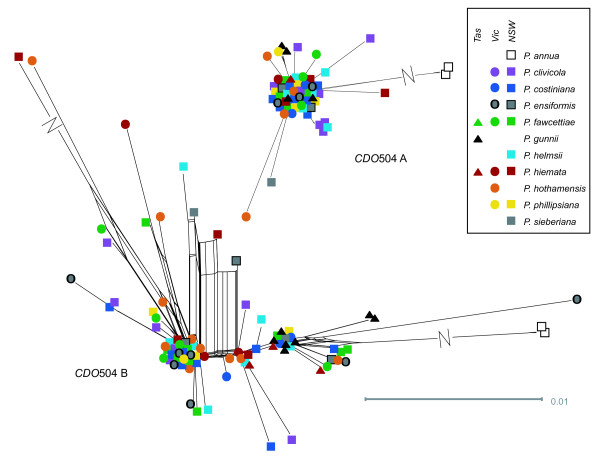
**Networks for the two *CDO*504 gene copies**. All networks created using the NeighbourNet algorithm in SplitsTree 4.11.3 using the EqualAngle layout option, from distance matrices created in Mega 5 (see text for details). Long splits are indicated by a break and not shown to scale.

**Figure 7:**
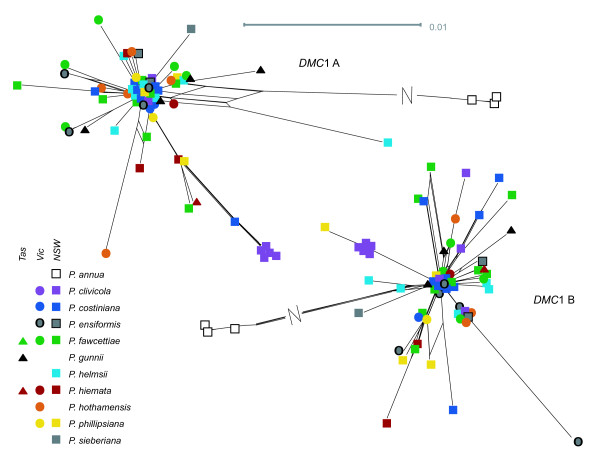
**Networks for the two *DMC*1 gene copies**. All networks created using the NeighbourNet algorithm in SplitsTree 4.11.3 using the EqualAngle layout option, from distance matrices created in Mega 5 (see text for details). Long splits are indicated by a break and not shown to scale.

As expected, the three *Poa annua *accessions formed a clear outgroup to the native Australian species for all gene regions (Figures 5, 6, 7), and most of the parsimony-informative sequence variation distinguished *P. annua *from the rest of the species (Table 1). Among the Australian species, a total of 23 parsimony-informative base changes and 7 informative insertion-deletion polymorphisms were detected in the chloroplast regions, and a further 38 base changes and 18 indels in the nuclear regions. By gene region, the chloroplast regions exhibited between 1.8 and 2.7% parsimony-informative variation among the Australian species, and the nuclear regions 1.1 to 5.2%.

**Table 1:** Sequence and alignment length, variation and substitution model chosen for each region sequenced

Region	Length range (bp)	Alignment length (bp)	Base changes (variable/parsimony-informative/parsimony-informative excluding outgroup)	Indels (parsimony-informative/parsimony-informative excluding outgroup)	% GC content	Nucleotide diversity, Π (total)	Tajima's D (overall/coding region)	Nucleotide substitution model
*rpl*32-*trn*L	426-448	470	46/19/11	8/2	26			TrN+I+G I = 0.69 G = 0.65

*rpo*B-*trn*C	419-430	449	37/27/3	8/5	30			F81+I I = 0.95

*trn*H-*psb*A	454-458	460	29/20/9	1/0	36			F81+G G = 0.01

*CDO*504 A	406-409	434	49/38/3	7/2	48	0.0050	-2.81***/-2.25**	F81

*CDO*504 B	389-432	467	86/56/17	12/8	48	0.010	-2.67***/-1.97*	TIM+G G = 0.59

*DMC*1 A	454-458	470	52/29/9	10/4	40	0.0024	-2.67***/(-1.76)	F81+G G = 0.67

*DMC*1 B	428-451	461	52/34/9	7/4	39	0.0032	-2.27**/(-0.56)	HKY+G G = 0.60 Ti/Tv = 0.87

For length range, only the Australian species are reported. For alignment length, the outgroup *Poa annua *is also included. Asterisks indicate *P*-values: * 0.01 <*P *< 0.05; ** 0.01 <*P *< 0.001; *** *P *< 0.001. Brackets indicate *P *> 0.05

No clear, consistent species differentiation was obvious from the genetic distance networks (Figures 5, 6, 7). *Poa gunnii *appeared somewhat distinct with the *rpl*32-*trn*L (Figure 5) and CDO504 B (Figure 6) markers, but was significantly differentiated only from two other species, and only in *rpl*32-*trn*L, by a pairwise Φ_PT _comparison (*P. gunnii *vs. *P. clivicola *Φ_PT _= 0.39, *P *< 0.05; *P. gunnii *vs. *P. costiniana *Φ_PT _= 0.44, *P *< 0.05 after Bonferroni correction). Most *P. clivicola *samples clustered together for both *DMC*1 A and *DMC*1 B (Figure 7), but not for the other gene regions. For *DMC*1 B, *P. clivicola *was significantly differentiated from *P. fawcettiae *(pairwise Φ_PT _- 0.22, *P *< 0.05 with Bonferroni correction) but not from any other species. In general, genotypes were shared by individuals of multiple species, and most species were not significantly differentiated by pairwise Φ_PT _comparisons.

### Inversional mutations in *trn*H-*psb*A

The *trn*H-*psb*A intergenic spacer produced a very different network from the other chloroplast regions (Figure 5). The rectangular shape was caused by two small inversions, a 6-bp region and a 2-bp region, which occurred in all four possible combinations with no species-specific patterns. We suggest this is an example of inversional mutation caused by hairpin secondary structure [36] (see predicted hairpin structure in additional file 1 - Figure S1). The appropriate hairpin structure was predicted using MFold [37], supporting this hypothesis. The same 6-bp inversion polymorphism was detected in alignments of multiple *Festuca *and *Lolium *sequences from GenBank. The 2-bp inversion region was extremely polymorphic across many of the Poaceae (see alignment in additional file 1 - Figure S1), and showed intraspecific variation in *Puccinellia*, *Festuca*, *Deschampsia *and *Oryza*. In fact, the entire putative hairpin region exhibited a high degree of variation.

### Geographic structure

Two of the chloroplast regions, *rpl*32-*trn*L and *rpo*B-*trn*C, showed evidence of spatial genetic structure. Significant dissimilarity was seen at the 200 to 300 and 600 to 800 km distance classes: this pattern disappeared when the Tasmanian samples were excluded, suggesting that the Tasmanian-mainland differentiation was responsible (see spatial autocorrelation plots in additional file 2 - Figure S2). For *DMC*1 A, low but significant dissimilarity was also seen at the 200 to 300 km distance class (*r *= -0.077, 95% CI = -0.027 to -0.105), which was strengthened when the Tasmanian samples were excluded (*r *= -0.089, 95% CI = -0.040 to -0.129). This was not explained by geographic source population (see Methods): no pair of populations was significantly differentiated (AMOVA *P *> 0.05 after Bonferroni correction).

At a smaller spatial scale, no genetic structure was detected using the nuclear markers, but significant positive structure was detected between 0 to 60 km for *rpl*32-*trn*L (falling to 0 to 30 km when Tasmanian samples were excluded) and 0 to 20 km for *rpo*B-*trn*C (see additional file 2 - Figure S2).

## Discussion

### Utility of next-generation sequencing

Phylogenetic analysis of polyploid species has typically been problematic, requiring large amounts of time and effort to sequence multiple gene copies in sufficient individuals using traditional Sanger sequencing and bacterial cloning [13,16,18]. However, high-throughput 'next-generation sequencing' of barcoded DNA mixtures is an affordable and successful solution to this problem. This effectively-clonal PCR method is financially viable for any type of mixed DNA sample. Next-generation methods involving sample barcoding have already been applied to a wide variety of bacterial, fungal and mixed environmental samples [for example 38, 39], and to multi-copy genes [40], but not to individual taxa containing a mixture of alleles, like polyploids or diploid heterozygotes, though the idea has been suggested [41].

In this project we have designed simple barcodes fused to the sequencing adaptors, a different method from those previously suggested [42]. Commercial sequencing facilities typically offer amplicon barcoding using the 10-bp 'multiplex identifiers' suggested by Roche [43]. These services can be expensive, and only 14 multiplex identifiers are available [43], limiting the number of samples. The barcode-adapter ligation protocol we describe here is relatively cheap and easy to implement. When sequencing an amplicon mixture, the level of barcode accuracy required is lower than that for other applications (genome sequencing projects, for example [44]) because individual reads with barcode sequencing errors will be detected and discarded in the alignment process. Thus, we suggest that simple 3 to 5 bp barcodes will be sufficient for most phylogenetic analyses. Their short length should prevent them from interfering with any stage of the amplification/sequencing process, especially if ligated after the initial PCR step. Longer barcodes could be designed to identify mixtures of more than the 61 individuals we examined here. Instead of attaching barcodes to the sequencing adapters as we did, it may be more sensible in some cases to synthesise PCR primers with built-in barcodes. In that case, barcoding power could be increased by using both barcoded forward and reverse PCR primers, but this would restrict the length of the sequenced regions, as each region would only be sequenced in one direction. Similar barcodes should also be suitable for other next-generation sequencing methods although distinguishing homeologues may be more difficult with shorter sequence reads such as those produced using the Illumina method. With some creativity and careful study design, appropriate barcodes can be developed to suit any situation.

Researchers accustomed to dealing with individual DNA chromatograms may feel overwhelmed by the large volume of data obtainable from a next-generation sequencing run. However, barcode deconvolution and quality control using the methods we describe here was quick. The entire workflow was completed in under an hour for each gene region. The time bottleneck actually occurred at the alignment and editing stage, which uses the same skills needed for 'traditional' molecular taxonomy, except that it is applied to larger numbers of sequences.

### Sequence success and repeatability

Despite efforts to mix equal amounts of DNA for each individual combination, there were significant differences between initial read numbers between different species. Since we were not interested in the relative abundance of different PCR products, but rather in sequencing all alleles that were present in each individual for each gene, this was not a problem. Drop-out rate was 12%, comparable to traditional sequencing methods using multiple markers [45]. Since we were able to sequence many more individuals than usual for a phylogenetic analysis, the occasional gene dropout for an individual did not affect our overall results.

Assuming 4 alleles were present at equal frequency in an individual, only 15 sequence reads were required to have a 95% chance of observing all 4 alleles (Figure 2) [32]. This was achieved for 54/64 individuals for CDO504 and 52/64 individuals for *DMC*1 (Figure 1). Much lower read counts were required to observe all distinct alleles if only two or three alleles were present per individual (Figure 2). This is in broad agreement with the 20 to 40+ clones recommended for sequencing multiple-copy nuclear genes using bacterial cloning [13]. However, we found significantly higher read numbers of copy B in both nuclear genes (copy B read no. 1.9 × copy A for CDO504, 1.2 × copy A for *DMC*1). This was despite efforts to reduce preferential amplification as suggested by Small *et al. *[13]. Five and seven sequence reads were needed to be confident of obtaining both copies in *DMC*1 and CDO504, respectively (Figure 2, red and blue lines), which was achieved in 58/64 samples for *DMC*1 and 54/64 samples for CDO504 (Figure 1).

We argue that the 454 sequencing method is more reliable than bacterial cloning for several reasons. First, cloning bias is avoided. Second, in Sanger sequencing, PCR errors that occur early in the reaction are propagated through later cycles, but this should not occur in next-generation sequencing because the sequencing reaction is performed on individual DNA molecules. Therefore sequencing errors are easier to detect in next-generation sequencing read alignments. Third, it is much easier to achieve the required numbers of sequence reads to obtain all alleles: in the past, researchers have often relied on far fewer clones than the 20 to 40+ recommended [19,22,46] and so missing alleles may be an issue in many phylogenetic studies, especially if preferential copy amplification is common.

PCR-based *in vitro *recombination is often observed in reactions with multiple templates [13,19]. We suspect that most of the PCR recombination we observed occurred in the initial PCR step [21], although it could have also occurred in the adapter-extension PCR step. However, the recombinant alleles were readily identified. *DMC*1 showed a higher proportion of recombinant sequences than did CDO504 (13.6% vs 2.9%). We have no clear explanation for this difference.

### Allopolyploidy in the Australian alpine *Poa *species

Two distinct gene copies were observed for each nuclear gene, differing by 4% and 8.5% (CDO504 and *DMC*1, respectively). As there was no evidence of recombination between them, we treated them as separately-evolving gene copies. This treatment is appropriate for polyploids. It is thought that homeologues do pair at meiosis in recent autopolyploids, but that most stable polyploid taxa become functionally diploid over time, meaning that gene copies from different ancestral genomes do not recombine [47]. As these *Poa *species seem to be functionally diploid, we would argue that they are relatively 'old' polyploids. This idea is supported by the fact that the same, identifiable gene copies were identified in all of the native Australian species examined, suggesting that they all evolved from a common allopolyploid ancestor.

Indeed, a previous study of some *Poa *species at the genus level found two distinct CDO504 copies of *P. sieberiana*, the only Australian species included in the study [21]. Our findings support this classification, with all of our Australian CDO504 copy A falling within sequence class A (as defined by Patterson et al.) and CDO504 copy B falling within sequence class C (Figure 4). Each of these sequence classes also contained *P. arctica*, *P. nervosa*, *P. fendleriana*, *P. iridifolia *and *P. arachnifera*, which are mostly restricted to North or South America (*P. arctica *is also found in the European Arctic) [48]. Two Australian/New Zealand species were also placed with some of these American species in an earlier study using chloroplast restriction site data [27]. The diploid progenitors of this Australian/American *Poa *group remain to be identified. The lack of resolution within these clades (Figure 4) means we cannot yet determine the geographic pattern of colonization, though it may be similar to that proposed for a related group of grasses, the New Zealand Loliinae, involving long-distance dispersal from North America to New Zealand via South America [49], followed by dispersal to Australia. We can state with confidence, however, that the Australian alpine species have not undergone polyploidization since colonization of the Alps, but that a polyploidization event took place before the Australian and the American species diverged.

Gene copies resulting from polyploidization often take on distinct functions or lose function [50]. Here we detected very few non-synonymous or synonymous mutations in the coding region of either *DMC*1 copy or CDO504 copy A, indicating that these three loci are probably still functional. Tajima's D was significantly negative in the introns of all four loci, indicating an excess of rare polymorphisms. This could have resulted from sequencing errors, a selective sweep or a recent population expansion. Sequencing error is unlikely to explain the large number of rare mutations we observed, because our method had a low (0.13%) error rate after quality control, and individual alleles were represented by many sequence reads. We suggest that population expansion is the most likely explanation, either expansion after the ancestral hybridization event, or expansion to colonize the 'new' environment of Australia. Sequencing this gene in the related New Zealand and American species would help to resolve this issue.

CDO504 copy B showed a different pattern, possessing several frameshift mutations that may render the protein product non-functional. This may indicate a loss of function in copy B, although every individual with an apparently non-functional allele also possessed a functional allele. There was also evidence of probable gene duplication in this copy: an allele with a 57-bp intron gap was present in most individuals, but as the gene dynamics were unclear, these alleles were excluded.

### Inversional mutation in *trn*H-*psb*A

Inversional mutation [36] explains the unusual rectangular network pattern in the *trn*H-*psb*A region and this mutation mechanism seems to have occurred throughout the grass family [see additional file 1 - Figure S1]. *Trn*H-*psb*A inversions have been reported for other angiosperm families [51-54] but not for grasses, probably because *trn*L-*trn*F and other loci are more widely used as phylogenetic markers in the grass family [55-57]. Also, grass phylogenies that have used this marker but only included one individual per species or genus [58,59] would necessarily fail to observe intra-taxon variation. *Trn*H-*psb*A has been proposed as a general DNA barcode region for land plants [60]. Our results support the argument that *trn*H-*psb*A is unsuitable as a barcode region because of its apparent propensity to inversion mutations [51].

### Species and geographic structure

The general pattern seen from both the chloroplast and the nuclear genes is one of extensive haplotype sharing between what are currently considered to be different species (Figures 5, 6, 7). The nuclear gene networks showed incongruence both with each other and with the chloroplast gene networks. Nuclear-organellar incongruence is common [18,20,46,61]; incongruence between nuclear genes is less widely reported, but does occur [22]. Both can result from incomplete lineage sorting or hybridization. Either of these processes could be occurring in the Australian alpine *Poa *species. They may well have resulted from a recent, rapid species radiation maintaining high effective population size, a typical scenario in incomplete lineage sorting [14,62]. Hybridization is also commonly reported within the genus *Poa *[27,28,63-65] and the entire grass family [9]. Experimental inter-species crosses between some of the species examined did produce viable offspring (Griffin, unpublished). Further work will attempt to distinguish between incomplete lineage sorting and hybridization as an explanation for the lack of species structure observed.

Some geographic pattern was revealed, with Tasmanian samples sharing a distinct clade in the *rpl*32-*trn*L and the CDO504 B networks, although some mainland samples also shared this clade in each case. The Tasmania-mainland differentiation was also detected in the spatial autocorrelation analysis, with significant genetic dissimilarity detected between pairs of individuals 500 to 900 or 700 to 900 km apart (in the markers *rpl*32-*trn*L and CDO504 A, respectively) that disappeared when the Tasmanian samples were removed from the analysis [see additional file 2 - Figure S2 A-B]. Although Tasmania was connected to the mainland as recently as 17,000 years ago at the last glacial maximum [66], similar divergence has been noted in a wide range of species [67-69]; in other species, genetic groups span the Bass Strait divide [70-72]. The low but significant spatial genetic differentiation at 200 to 300 km noted with the *DMC*1 copy A marker was not explained by geographic clusters reflecting the source mountain range or broad geographic area and may be a chance result due to sampling bias.

On the local scale, fairly strong spatial genetic structure was detected using two of the chloroplast markers. *Rpl*32-*trn*L found significantly similar genotypes up to 60 km apart, which decreased to 30 km apart when the Tasmanian samples were excluded [see additional file 2 - Figure S2 E-F]. *Rpo*B-*trn*C, which had less genetic variation overall (Table 1), revealed spatial genetic similarity in neighbourhoods of up to 20 km with or without the Tasmanian samples [see additional file 2 - Figure S2 G-H]. No significant local-scale genetic structure was detected with the nuclear markers. Because the chloroplast is maternally inherited in most angiosperms, these results suggest a smaller neighborhood for seed dispersal than for pollen dispersal. The Australian alpine zone occurs across small, relatively isolated 'sky islands', and strong spatial genetic structure has been detected in Australian alpine and montane lizards [70,73,74]. However, it is perhaps not surprising that these alpine grasses lack spatial genetic structure with nuclear markers: they are wind-pollinated, and high population connectivity has been documented in one *Poa *species across mountaintops approximately 8 kilometres apart [75].

## Conclusions

We developed a novel method of barcoding amplicon mixtures from polyploid individuals for 454 sequencing. We successfully sequenced multiple nuclear genes, each with multiple copies and multiple alleles, and also sequenced three single-copy chloroplast regions, all in 64 individuals representing 11 species. This method had high replicability, a low error rate after quality control and a low rate of missing data (88% of the 320 gene/individual combinations produced sequence reads). This method is cheaper than and at least as reliable as bacterial cloning. It could be applied to any experiment involving sequencing of amplicon mixtures.

We applied this method to a group of sympatric Australian alpine *Poa *species, which were discovered to have an allopolyploid ancestor in common with a group of American *Poa *species. Alleles and haplotypes were shared extensively between the Australian 'species' and little inter-species differentiation was detected, though the Tasmania and mainland samples were somewhat distinct. Significant spatial genetic structure was detected at <100 km spatial scales using chloroplast but not nuclear markers, indicating smaller seed than pollen neighborhoods. More work is necessary to distinguish incomplete lineage sorting from hybridization in this seemingly recently-evolved group, but we can recommend that the current taxonomy be re-examined, as we found little evidence to support current species concepts.

## Methods

### Sampling and DNA extraction

Herbarium specimens were examined at the Australian National Herbarium, Canberra, Australia (CANB). Eleven species were chosen, with a total of 61 specimens sampled (Table 2). Specimens sampled had been collected later than 1970, identified to the species or variety level by experienced taxonomists, and with large amounts of well-preserved leaf tissue from a single plant. A small amount of leaf tissue (1/2 to 5 leaves depending on plant size) was removed. In the laboratory, approximately 20 mg of leaf tissue was added to a 1.5 ml Eppendorf tube with two glass beads (3 mm diameter). DNA was extracted using the DNeasy Plant Mini Kit (Qiagen, Hilden, Germany) following the manufacturer's protocol, grinding leaf tissue in a Mixermill (Retsch MM300) and eluting in a total volume of 200 μl. Duplicate samples of three herbarium specimens were included from the DNA extraction stage onwards, bringing the total number of samples to 64.

**Table 2:** Ecological association and herbarium accessions used for each *Poa *species

Species	Ecological association	Herbarium accessions (all CANB)		
		
		Victoria	New South Wales	Tasmania
Poa annua* (5)	Subalpine, montane, elsewhere		312513, 343723, 407148	

*P. clivicola *(8)	Alpine, subalpine		228786, 228805, 329728, 438760, 647153, 7901040, 8702739, 8906309	

*P. costiniana *(8)	Alpine	402908**	228898, 505348, 543047**, 619540, 619578, 8101033, 9707880	

*P. ensiformis *(5)	Subalpine, montane	48084, 67340, 363091	645156, 8906370	

*P. fawcettiae *(10)	Alpine	67067, 488116, 9501151	228865, 228879, 230925, 345640, 505340, 505353, 505353	

*P. gunnii *(5)	Alpine, subalpine	Not present	Not present	195943, 341316, 402406, 8904303, 9215410

*P. helmsii *(6)	Alpine, subalpine		68026, 228839, 312845, 644862, 8316158, 9301326	

*P. hiemata *(5)	Alpine	8500047	343721, 506031, 7705690	9012247

*P. hothamensis *var. *hothamensis *(6)	Alpine, subalpine	67341, 207890**, 207886, 207893, 409990	Not present	Not present

*P. phillipsiana *(6)	Alpine, subalpine	67342, 207889	615444, 651518, 8413383	

*P. sieberiana *var. *sieberiana *(1)	Alpine, subalpine, montane, elsewhere		780260	

Herbarium accession numbers are listed by Australian state of origin. Total number of accessions used is given in brackets after the species name.

* indicates the introduced species *Poa annua*, used as an outgroup

** indicates specimens that were replicated from the DNA extraction stage onwards to check for replicability

### Initial PCR and amplicon pooling

The program Primer3 [76] was used to design primers for amplifying regions 400 to 450 bp in length from the nuclear regions *DMC*1 and CDO504, and the following chloroplast regions: *rpl*32-*trn*L, *rpo*B-*trn*C and *trn*H-*psb*A. The maximum sequence length for this study was limited to approximately 500 bp as recommended by Roche [43]. Primers were designed based on initial sequence alignments gained from a trial panel of 25 individual herbarium specimens [see additional file 3 - Table S1]. These had been obtained using primers designed by Patterson *et al. *[21] (CDO504), Petersen and Seberg [77] (*DMC*1, primers TDMC1e10 and TDMC1e15R), Shaw *et al. *[78] (*trn*H-*psb*A and *rpo*B-*trn*C), and Shaw *et al. *[79] (*rpl*32-*trn*L) (results not reported here). For all PCR amplifications, 2 × BIO-X-ACT™ Short Mix (Bioline, London, England) containing enzyme, buffer, dNTPs and 2.0 mM MgCl_2 _was used in 20 μl reactions. Primers (0.8 μM each primer), template (2 μl extracted DNA for nuclear amplifications, 2 μl 1/10-diluted extracted DNA for chloroplast amplifications) and MgCl_2 _[see additional file 3 - Table SI] were added and the volume made up to 20 μl with 2 × PolyMate Additive (Bioline, London, England). The basic PCR protocol was as follows: 96°C for 5 minutes; 40 cycles of 96°C for 40 seconds, 50°C for 60 seconds, 72°C for 40 seconds; 72°C for 5 minutes; 15°C hold. Variations from this protocol for each marker are shown in additional file 3 - Table S1. For the nuclear genes, three PCR reactions were pooled for each sample/marker combination to avoid preferential amplification of particular alleles, as recommended by Small *et al. *[13]. Amplification products were quantified by agarose gel electrophoresis and comparison to a ladder (HyperLadder™ II, Bioline, London, England). For the chloroplast regions, 25 ng of each PCR product was pooled for each individual. 100 ng of each nuclear region was added, because we wanted to represent each of the four possible amplicons equally in the final mixture.

### Barcoded amplicon library creation

**Figure 8:**
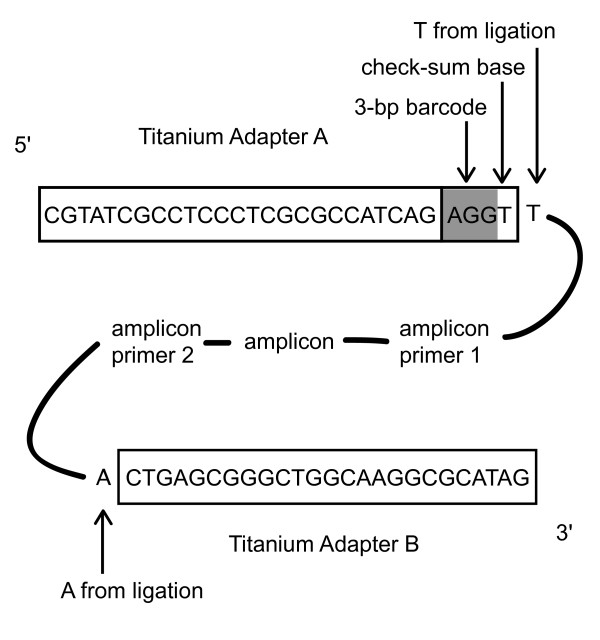
**Diagram of barcoded amplicon**. The amplicon of interest, flanked by forward and reverse PCR primer sequences, was subjected to ligation of the Titanium Adapter B and Adapter A + barcode sequence, producing an oligonucleotide of the form shown. One out of 64 barcode + check-sum base sequences is represented here. Initially, the Adapter A oligonucleotide used was 6 bp shorter at the 5' end. It was extended by PCR to match the Titanium sequence shown here, as described in the text.

To avoid synthesizing a composite amplicon primer-barcode-adapter fusion primer, for every gene × individual combination [42,43], which would have been prohibitively expensive, a ligation reaction was performed to add barcode sequences to the pooled amplicons. The barcodes comprised the GS FLX Adapter A (5'--CGTATCGCCTCCCTCGCGCCATCAG--3') with a 3-bp barcode sequence at the 3' end as in [44] followed by a checksum base and an extra T to facilitate sticky-ended ligation (Figure 8). The checksum base was included to detect sequencing errors in the barcode region, such that the barcodes occupied only 64/256 possible 4-bp-long combinations of the four bases. The reverse complement of this Adapter A-barcode oligonucleotide was also present. Adapter B (5'--CTGAGCGGGCTGGCAAGGCGCATAG--3') was a non-barcoded, non-biotinylated oligonucleotide identical to that used in the current GS FLX Titanium Lib-L sequencing chemistry [80]. To make the barcodes double-stranded, equimolar mixtures in ligase buffer (BioLine, London, England) of the forward and reverse-complement oligonucleotides (each 20 μM) were subjected to 95°C for 3 minutes and allowed to cool to room temperature.

Barcodes were ligated onto the amplicons using a standard ligation reaction as follows. The amplicon mixture for each individual was purified using an Illustra GLX PCR DNA and Gel Band Purification Kit (GE Healthcare, Little Chalfont, UK, 2008 version). The purified product was quantified using a NanoDrop 1000 Spectrophotometer (Thermo Scientific, Wilmington, DE, USA).100 ng of the pooled amplicon mixture, double-stranded Adapter A-barcode and double-stranded Titanium Adapter B (0.25 μM each), 5 U T4 polynucleotide kinase (New England Biolabs, Ipswich, MA, USA), 20 cohesive end units T4 DNA ligase (New England Biolabs, Ipswich, MA, USA) and 10 × T4 ligase buffer were combined in a 40 μl ligation reaction. This mixture was subjected to 37°C for 1 hour, followed by 10 cycles of 22°C for 1 hour, then placed at 14°C for 1 hour, and 65°C for 20 minutes before being brought to room temperature. The desired ligation product was flanked by the Adapter A-barcode oligonucleotide on one end and the double-stranded Titanium Adapter B on the other end (Figure 8). Some products would have formed with either Adapter A-barcode or Adapter B on both ends; these would be removed automatically later in the process, in the Enrichment step described in the Roche GS FLX Titanium emPCR Method Manual [80]. To remove excess adaptors, all ligations were pooled in groups of 5 (10 μl each ligation) and the resulting mixtures were purified using a PCR Purification Kit (Scientifix, Clayton, Victoria, Australia).

To check the success of the ligations for two samples, the ligation products were ligated into a pGEM-T Easy Vector (Promega, Madison, WI, USA) after addition of an A-overhang and used to transform JM-109 competent cells according to the manufacturer's protocol. Three clones were sequenced for each sample to check whether the original ligation had occurred as desired, using commercial ABI-3730XL capillary sequencing (Macrogen Inc, Geumcheon-gu, Seoul, Korea). Sequences were examined using Sequencher 4.7 (GeneCodes Corporation, Ann Arbor, MI, USA).

Our barcodes had been designed to match the 2007 GS FLX chemistry, so the Adapter A sequence was 6 bp shorter than the 2010 Titanium Adapter A (which was to be used in the actual sequencing reaction). To overcome this problem, after the adapter ligation step, we performed three replicate PCR reactions for each mixture using Titanium Adapter A as the forward primer and Titanium Adapter B as the reverse primer. This reaction extended the barcode end of the samples an extra 6 bp so that they were of the form: 5' - Titanium A adapter - barcode - checksum base - T - amplicon - A - Titanium B adapter - 3', to match exactly with the current Titanium chemistry (Figure 8). PCR protocol was as follows: 96°C for 5 minutes; 10 cycles of 96°C for 30 seconds, 53°C for 30 seconds, 72°C for 45 seconds; 72°C for 5 minutes; 15°C hold. By designing barcode oligonucleotides to match exactly the current sequencing chemistry, researchers should be able to avoid this step.

After the PCR extension step, the amplicon mixtures were pooled in equal amounts. The resulting barcoded amplicon library (approximately 630 ng of DNA) was purified again using the PCR Purification Kit (Scientifix, Clayton, Victoria, Australia) to remove PCR primers and sequenced using the GS FLX Titanium chemistry (Lib-L; Roche, Basel, Switzerland). A ¼ plate run was performed following appropriate quality control and sample preparation carried out by the Australian Genome Research Facility (AGRF), Brisbane, Australia.

### Barcode deconvolution and quality control

Sequence (.fasta) and quality (.qual) files obtained from the sequencing run were imported into the public Galaxy platform, a free online bioinformatics interface available at http://main.g2.bx.psu.edu/[81]. The full dataset was divided into gene regions by regular expression (REGEX) matching the amplicon primer sequences. Since this work was carried out, 'clip adapter' and 'barcode splitter' tools have become available in the FASTX-Toolkit (http://hannonlab.cshl.edu/fastx_toolkit/) implemented in Galaxy, which may simplify the procedure further. Individual barcodes were then detected using R [82] [see additional file 4 - Supplementary Text].

**Figure 9:**
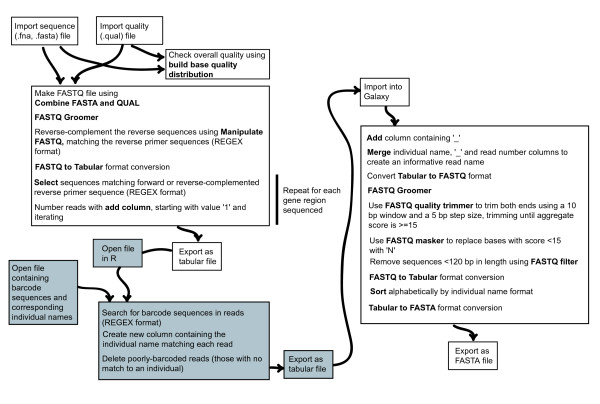
**Barcode deconvolution and quality control workflow**. White boxes indicate commands carried out in Galaxy (http://main.g2.bx.psu.edu/), with tool names shown in bold. Grey boxes indicate commands carried out in R. R code and examples of REGEX matching are available in additional file 3 (Supplementary Text).

After this barcode deconvolution, quality control was performed to trim low-quality read ends, mask unreliable base calls and remove short sequences. This workflow is shown in detail in Figure 9. A subset of *rpl*32-*trn*L reads was examined in R to check the accuracy of the quality control procedure and to estimate the frequency of barcode failure. Finally, a FASTA file containing all good-quality reads for each gene region, named by the source individual, was exported for alignment and editing.

### Sequence alignment and editing

Sequences were aligned using Sequencher 4.7 [83] for each combination of individual and gene. Alignments were edited manually to remove adapters, PCR primers and bases that had been added during the ligation process. For the chloroplast regions, a single consensus product was expected for each individual. For the nuclear regions, distinct alleles were identified as differing consistently by at least two base positions in >80% of reads in the alignment. A separate alignment was then edited manually for each allele.

Sequencing error rate was estimated by counting the single-base variants in 25 of the *rpl*32-*trn*L alignments (>20,000 bases). Poor-quality (<15) bases were excluded from error estimation as they had been previously masked with Ns. Length variation was also excluded.

Apparent alleles that actually resulted from PCR recombination were detected using two criteria. First, these apparent alleles occurred in a minority of reads for that particular individual/gene combination (usually <5%). Second, one end of the recombinant sequence matched one common allele and the other end matched a different common allele when all three were examined in a contig. A subset of the alleles identified as recombinant were checked using the BootScan method [84] in RDP3 [33].

Once a suitable sequence alignment was produced, indels were coded. Apparent length variation in mononucleotide repeats of >5 bp was not scored, as there was often no clear consensus between sequence reads at such regions. Indels that were not phylogenetically informative (that is only occurred in one individual) were also not scored, as they may have resulted from PCR errors. All other indels were coded as 'a' or 'c' and added to the end of the sequence alignment, so weighting them as transversions.

### Data analysis

For each sequence alignment, the best substitution model was chosen by AIC value comparison using Modeltest 3.7 [85] implemented in PAUP* v4b10 [86]. A pairwise genetic distance matrix was then calculated using Mega 5 [87] with substitution model parameters as close to those chosen in Modeltest as possible. This distance matrix was followed to make a network in SplitsTree 4.11.3 [88], using the NeighbourNet method and the EqualAngle display option.

For the chloroplast regions, the same pairwise distance matrix was used for investigating species and geographic genetic structure. For the nuclear regions, an uncorrected p-distance matrix made in Mega 5 was converted to an individual distance matrix with Pofad 1.03 [89]. For all seven gene regions, the following analysis was performed in GENALEX 6.3 [90]. First, *Poa annua *sequences and any species with only a single representative individual were removed. Either species or broad geographic groups (Tasmania, western Victoria, north-eastern Victoria, Kosciuszko Main Range (eastern NSW), Australian Capital Territory, NSW lowlands) were coded as populations. An analysis of molecular variance (AMOVA) was performed with 999 permutations and both pairwise and overall Φ_PT _output. Second, the genetic distance matrix was recoded as a single population, and a geographic distance matrix was calculated from the latitude/longitude of sample collection listed on the herbarium record of each individual. A spatial autocorrelation analysis was then performed using ten even distance classes of 100-km intervals, 999 permutations and 999 bootstrap resampling steps. It was repeated for each region excluding all Tasmanian samples. A second analysis was performed with 20-km distance intervals for gene regions that showed spatial genetic structure in the first analysis.

Representative CDO504 copy A and B sequences were chosen that covered the sequence diversity observed in this study. CDO504 sequences for other *Poa *species were downloaded from GenBank [30]. All sequences were aligned manually using Sequencher 4.7 without coding insertion-deletion polymorphism. The best model of nucleotide evolution was again chosen using Modeltest 3.7, run in PAUP*. Bayesian analysis was then performed on the alignment in BEAST 1.6.1 [91], running 10,000,000 MCMC generations and logging every 1,000 generations, with the following departures from the default priors: constant population size; GTR+G substitution model with starting substitution matrix and gamma parameter values chosen using ModelTest. A maximum clade credibility tree was then calculated using TreeAnnotator after removing the first 1,000,000 generations as burnin. A maximum parsimony analysis was also run on the alignment using 200 iterations of the ratchet provided in PaupRat [92] implemented in PAUP*, with random-addition searches with TBR branch swapping. One thousand bootstrap repetitions were then performed, using 50 random sequence addition heuristic searches and TBR branch swapping.

## Abbreviations

G: Gamma parameter (in a nucleotide substitution model); GTR: Gamma Time-Reversible; I: proportion Invariant sites (in a nucleotide substitution model); Indel: insertion-deletion mutation; PCR: Polymerase Chain Reaction; SNP: Single-Nucleotide Polymorphism; TBR: Tree Bisection-Reconnection.

## Authors' contributions

PCG performed the molecular lab work, developed the barcode deconvolution and quality control method, carried out the data analysis, and drafted the manuscript. CR developed the sample barcode design, participated in the study design, suggested some tests of the sequencing method, annotated nuclear regions and helped to draft the manuscript. AAH participated in the study design and helped to draft the manuscript. All authors read and approved the final manuscript.

## Supplementary Material

Additional file 1 **Figure S1 - Alignment of partial trnH-psbA spacer region showing insertional mutation across the Poaceae**. The predicted hairpin structure is shown in the upper panel, with conserved regions involved in hairpin binding colored as per the alignment in the lower panel. Species names are shaded according to subfamily: Arundinoideae (pink), Bambusoideae (light blue), Chloridoideae (green), Ehrhartoideae (Yellow), Panicoideae (dark blue), Pooideae (red), uncertain (grey). One Liliaceae sequence is included, outlined in black. Click here for file

Additional file 2 **Figure S2 - Spatial genetic autocorrelation plots for the chloroplast markers**. The two markers that revealed spatial genetic structure are shown: *rpl*32-*trn*L (A-B, E-F) and *rpo*B-*trn*C (C-D, G-H). Analyses were repeated including (A, C, E, G) and excluding (B, D, F, H) the Tasmanian samples, and for large-scale (A-D) and small-scale (E-H) distances. Solid black line joins the mean *r *values for each distance class, with 95% CI shown by the error bars (determined by 999 bootstrap resampling repeats). The dotted red lines bound the 95% CI about the null hypothesis (determined by 999 permutations). Click here for file

Additional file 3 **Table SI - Primers and PCR details for the regions amplified**. Table showing primer sequences and variation from the PCR protocol described in the text for each gene region amplified. Click here for file

Additional file 4 **Supplementary Text - Detailed instructions for barcode deconvolution in R**. Text file including R code. Click here for file
