# Supplementary material for: A next-generation sequencing method for overcoming the multiple gene copy problem in polyploid phylogenetics, applied to Poa grasses
Source: BMC Biol. 2011 Mar 23;9:19. doi: 10.1186/1741-7007-9-19 (PMC3078099; doi:10.1186/1741-7007-9-19)
Supplement: Additional file 1 — Figure S1 - Alignment of partial trnH-psbA spacer region showing insertional mutation across the Poaceae. The predicted hairpin structure is shown in the upper panel, with conserved regions involved in hairpin binding colored as per the alignment in the lower panel. Species names are shaded according to subfamily: Arundinoideae (pink), Bambusoideae (light blue), Chloridoideae (green), Ehrhartoideae (Yellow), Panicoideae (dark blue), Pooideae (red), uncertain (grey). One Liliaceae sequence is included, outlined in black. [file 1741-7007-9-19-S1.PDF]

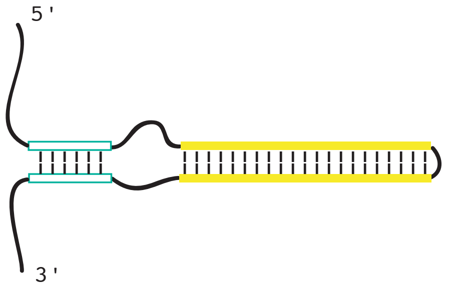


| | |
|---|---|
| Phragmites_japonicus_FJ766222 | AATTCAGAATAGAAAGAT-TCAGAATAAA--------CAA--------------AGAAATACCCAATATCTTGTTGGAACAAGATATTGGGTATTTCTGGCTTTCCTTCCTTTA |
| Chimonocalamus_pallens_FJ644255 | AATTCAGAATAGAAAGAT-TCAAAATAAAAAAAA---CAA--------------AGAAATACCCAATATCTTGTTCCAGCAAGATATTGGGTATTTCTGGCTTTCCTTCCTTCA |
| Pleioblastus_argenteostriatus_FJ644262 | AATTCAGAATAGAAAGAT-TCAAAATAAAAAAAA---CAA--------------AGAAATACCCAATATCTTGCTGGAACAAGATATTGGGTATTTCTGGCTTTCCTTCCTTCA |
| Sasa_borealis_FJ766224 | AATTCAGAATAGAAAGAT-TCAAAATAAAAAAAAAAAACAA--------------AGAAATACCCAATATCTTGCCGGAACAAGATATTGGGTATTTCTGGCTTTCCTTCCTTCA |
| Eragrostis_ferruginea_FJ766208 | AATTCAGAATAGAAAGAT-TCAGAATAAA--------CAA--------------AGAAATACCCAATATCTTGTTGAAACAAGATATTGGGTATTTCCGGCTTTCCTTCCTTTA |
| Muhlenbergia_huegelii_FJ766217 | AATTCAGAATAGAAAGAT-TCAGAATAAATAA---------------------AGAAATACCCAATATCTTGTTCCAACAAGATATTGGGTATTTCCGGCTTTCCTTTCTTTA |
| Oryza_latifolia_GU575283 | AATTCAGAATAGAAAGAT-TCAAAATAAAAAAAAAAA-----------------AGAAATACCCAATATCTTGCTGAAACAAGATATTGGGTATTTCTAGCTTTCCTTTCTTCA |
| Oryza_longistaminata_GU575243 | AATTCAGAATAGAAAGAT-TCAAAATAAAAAAAAAAAA----------------AGAAATACCCAATATCTTGCTTCAGCAAGATATTGGGTATTTCTAGCTTTCCTTTCTTCA |
| Oryza_sativa_Indica_GU575239 | AATTCAGAATAGAAAGAT-TCAAAATAAAAAAAAAAA-----------------AGAAATACCCAATATCTTGCTGAAGCAAGATATTGGGTATTTCTAGCTTTCCTTTCTTCA |
| Oryza_sativa_Indica_GU575241 | AATTCAGAATAGAAAGAT-TCAAAATAAAAAAAAAAAA----------------AGAAATACCCAATATCTTGCTTCAGCAAGATATTGGGTATTTCTAGCTTTCCTTTCTTCA |
| Arundinella_hirta_FJ766195 | AATTCAGAATAGAAAGAT-TCAGAATAAA--------CAA--------------AGAAATACCCAATATCCTGTTGGAACAAGATATTGGGTATTTCCGGCTTTCCTTCCTTCA |
| Digitaria_ciliaris_FJ766202 | AATTCAGAATAGAAAGAT-TCAGAATAAA--------CAA--------------AGAAATACCCAATATCCTGTTGGAACAAGATATTGGGTATTTCTGGCTTTCCTTCTTTCA |
| Setaria_viridis_FJ766226 | AATTCAGAATAGAAAGAT-TCAGAATAAA--------CAA--------------AGAAATACCCAATATCCTGTTGGAACAAGATATTGGGTATTTCTGGCTTTCCTTCTTTCA |
| Alopecurus_japonicus_FJ766193 | AATTCAGAATAGAAAGAT-TCAGAATAAA--------CAA--------------AGAAATACCCAATATCTTGTTGGAACAAGATATTGGGTATTTCTAGCTTTTCTTTCTTCA |
| Cinna_latifolia_FJ766192 | AA-------TAGAAAGAT-TCAGAATAAA--------CAA--------------AGAAATACCCAATATCTTGTTGGAACAAGATATTGGGTATTTCTAGCTTTTCTTTCTTCA |
| Dactylis_glomerata_FJ395491 | AATTCAGAATAGAAAGAT-TCAGAATAAA--------CAAAGGATTCAAAATAAAGAAATACCCAATATCTTGTTAGAACAAGATATTGGGTATTTCTAGCTTTTCTTTCTTCA |
| Diarrhena_fauriei_FJ766200 | AATTCAGAATAGAAAGAT-TCAGAATAAAAAAAA--CAA--------------AGAAATACCCAATATCTTGCTGGAACAAGATATTGGGTATTTCTGGCTTTTCTTTCTTCA |
| Elymus_tsukshiensis_FJ766190 | AATTCTGAATAGAAAGAT-TAAGAAGAAA--------AAAAAGATTCAAAAGACAGAAATACCCAATATCTTGTTCTAGCAAGATATTGGGTATTTCTGTCTTTTCTTTCTTCA |
| Festuca_roemeri_DQ369786 | AATTCAGAATCTTTCTATATCTGAATAAA--------CAA--------------AGAAATACCCAATATCTTGTTCTAGTAAGATATTGGGTATTTCTAGCTTTTCTTTCTTCA |
| Festuca_rubra_DQ369760 | AATTCAGAATAGAAAGAT-TCTGAATAAA--------CAA--------------AGAAATACCCAATATCTTA-----TCAAGATATTGGGTATTTCTAGCTTTTCTTTCTTCA |
| Holcus_lanatus_FJ395493 | AATTCAGAATAGAAAGAT-TCAGAATAAA--------CAA--------------AGAAATACCCAATATCTTGCTAGAACAAGATATTGGGTATTTCTAGCTTTTCTTTCTTCA |
| Leymus_sabulosus_EF485869 | AATTCTGAATAGAAAGAT-TAAGAAAAAAAAAAA-------------------AGAAATACCCAATATCTTGCTAGAACAAGATATTGGGTATTTCTGTCTTTTCTTTCTTCA |
| Lolium_sp_FJ766215 | AATTCAGAATCTTTCTAT-TCTGAATAAA--------CAA--------------AGAAATACCCAATATCTTGCTAGAACAAGATATTGGGTATTTCTAGCTTTTCGTTCTTCA |
| Phaenosperma_globosa_FJ766218 | AATTCAGAATAGAAAGAT-TCAGAATAAAAAAA----CAA--------------AGAAATACCCAATATCTTGCTGGAACAAGATATTGGGTATTTCTGACTTTCCTTTCCTCA |
| Phalaris_arundinacea_FJ766221 | AATTCAGAATAAA------------------------CAA--------------AGAAATACCCAATACCTTGTTTCAACAAGATATTGGGTATTTCTGGCTTTTCTTTCTTCA |
| Poa_clivicola_ACT_102 | AATTCAGAATAGAAAGAT-TCAGAATAAA--------CAA--------------AGAAATACCCAATATCTTGTTCCAACAAGATATTGGGTATTTCTAGCTTTTCTTTCTTCA |
| Poa_fawcettiae_NSW_134 | AATTCAGAATCTTTCTAT-TCTGAATAAA--------CAA--------------AGAAATACCCAATATCTTGTTCCAACAAGATATTGGGTATTTCTAGCTTTTCTTTCTTCA |
| Poa_pratensis_FJ766223 | AATTCAGAATAGAAAGAT-TCAAAATAAAATAA--------------------AGAAATACCCAATATCTTGTTCCAACAAGATATTGGGTATTTCTAGCTTTTCTTTCTTCA |
| Streptogyna_americana_FJ644272 | AATTCAGAATAGAAAGAT-TCAGAATAAAAAAAA-------------------AGAAATACCCAATATCTTGCTGGAACAAGATATTGGGTATTTCTGGCTTTCCTTCCTTCA |
| Bomarea chiriquina GQ429131 | AATTCAGAATAGAAAGAT-TCAGAATAAA--------CAA--------------AGAAATACCCAATATCTTGTTGGAACAAGATATTGGGTATTTCTTGCTTTTCTTTCTTCA |
