# Supplementary figures and images for: A next-generation sequencing method for overcoming the multiple gene copy problem in polyploid phylogenetics, applied to Poa grasses
Source: BMC Biol. 2011 Mar 23;9:19. doi: 10.1186/1741-7007-9-19 (PMC3078099; doi:10.1186/1741-7007-9-19)

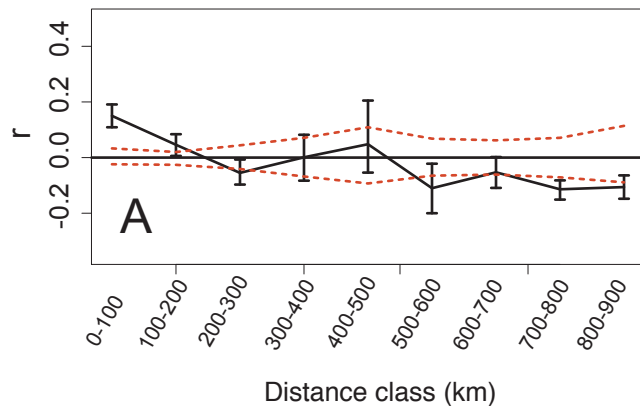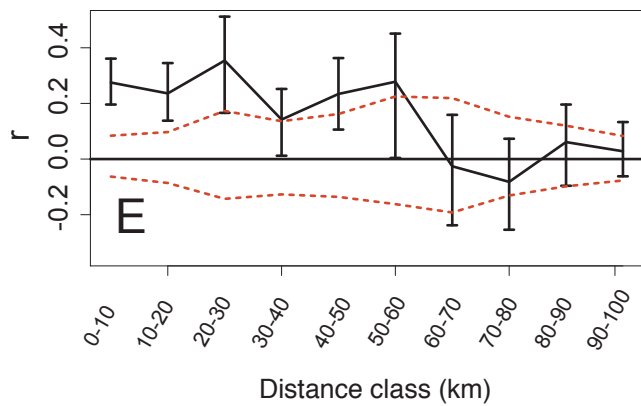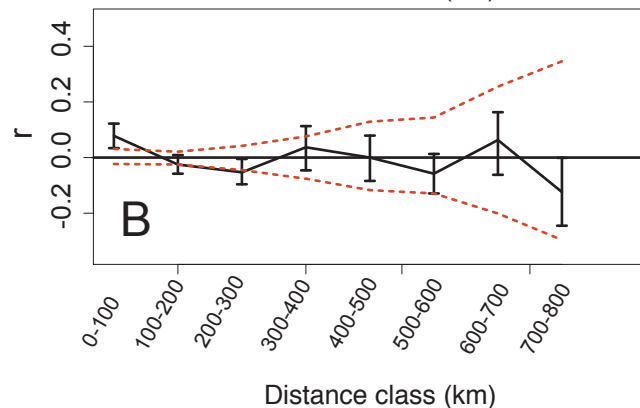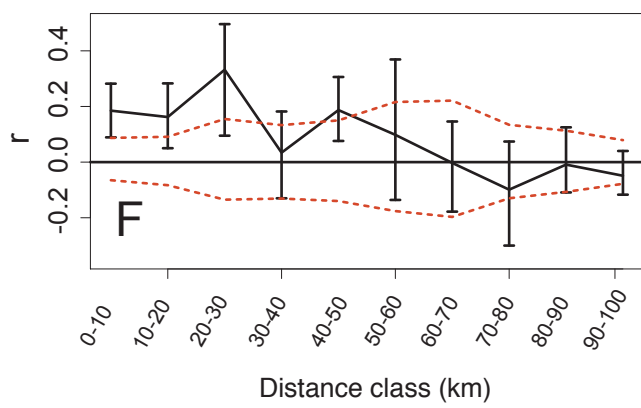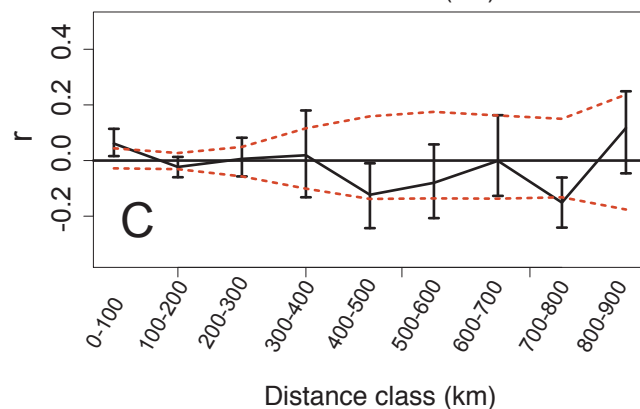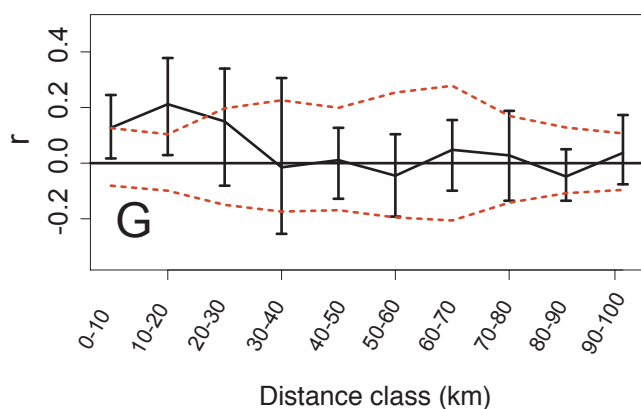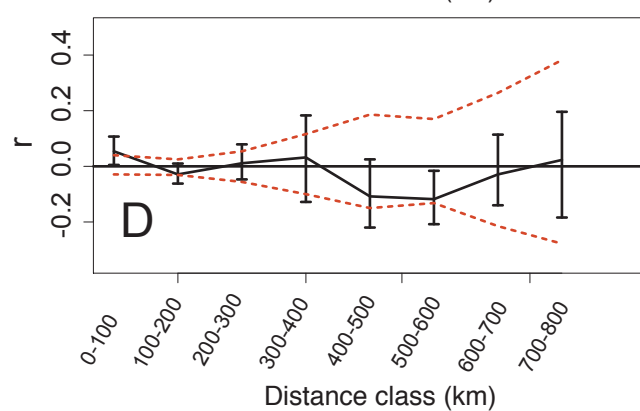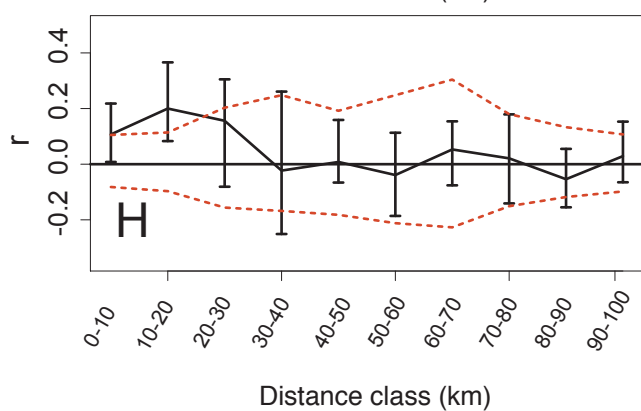

Supplement: Additional file 2 — Figure S2 - Spatial genetic autocorrelation plots for the chloroplast markers. The two markers that revealed spatial genetic structure are shown: rpl32-trnL (A-B, E-F) and rpoB-trnC (C-D, G-H). Analyses were repeated including (A, C, E, G) and excluding (B, D, F, H) the Tasmanian samples, and for large-scale (A-D) and small-scale (E-H) distances. Solid black line joins the mean r values for each distance class, with 95% CI shown by the error bars (determined by 999 bootstrap resampling repeats). The dotted red lines bound the 95% CI about the null hypothesis (determined by 999 permutations). [file 1741-7007-9-19-S2.PDF]
