# Supplementary material for: A next-generation sequencing method for overcoming the multiple gene copy problem in polyploid phylogenetics, applied to Poa grasses
Source: BMC Biol. 2011 Mar 23;9:19. doi: 10.1186/1741-7007-9-19 (PMC3078099; doi:10.1186/1741-7007-9-19)
Supplement: Additional file 3 — Table SI - Primers and PCR details for the regions amplified. Table showing primer sequences and variation from the PCR protocol described in the text for each gene region amplified. [file 1741-7007-9-19-S3.DOC]

**Table SI**: Primers and PCR details for the regions amplified.

| Region | Forward primer (5’-3’) | Reverse primer (5’-3’) | Amount template | [MgCl2] | Annealing temp | Annealing time | Extension time | No. cycles |
| --- | --- | --- | --- | --- | --- | --- | --- | --- |
| *rpl*32-*trn*L | CAAACCCCTCTGATATCTGCT | CACTGCTTGAAAGAGTTGGAA | 2 uL 1/10 diln | 2.5 mM | 50ºC | 60 s | 90 s | 45 |
| *rpo*B-*trn*C | CGAGCGAAAATCGAGAAAAG | TAATGCTGTTTGGGTCTGGA | 2 uL 1/10 diln | 2.5 mM | 50ºC | 60 s | 90 s | 50 |
| *trn*H-*psb*A | CGAATCCTTTTGTAGCTCATCA | GCATGAACGTAATGCTCACA | 2 uL 1/10 diln | 2.5 mM | 50ºC | 60 s | 90 s | 40 |
| *DMC*1 | CGATCACTGGAACAAGGACA | CTTTCAGGCTTCTGGTAGGC | 2 uL | 2.5 mM | 50ºC | 60 s | 40 s | 40 |
| *CDO*504 | CCCTCTACCATCTCGTCCAC | ATGAATGCAAAGCCACCAAT | 2 uL | 2.0 mM | 55ºC | 60 s | 40 s | 42 |
