# Supplementary material for: A next-generation sequencing method for overcoming the multiple gene copy problem in polyploid phylogenetics, applied to Poa grasses
Source: BMC Biol. 2011 Mar 23;9:19. doi: 10.1186/1741-7007-9-19 (PMC3078099; doi:10.1186/1741-7007-9-19)
Supplement: Additional file 4 — Supplementary Text - Detailed instructions for barcode deconvolution in R. Text file including R code. [file 1741-7007-9-19-S4.DOC]

**Supplementary Text:** Detailed instructions for barcode deconvolution in R.

The file 'Readnos.csv' will need to be converted from a tabular format to a comma-delimited .csv format after output from Galaxy. It should contain two columns.

Column 1: Read number, ordered. Column 2: Read sequence ('Seq')

e.g.

No,Seq

1,TCCCATCGAGCGAAAATCGAGAAAAGAGTAAGTATTCATCCACGTTTTCTTACGAAAACCCCTTTTATTTTAATATAATTCTACTTACTTTTTCCTAATCTTTTAAAAAATTTAATT…

2,CCACTC...

3,TCGAGC...

...

The file 'IDs.csv' should contain two columns.

Column 1: Individual name. Column 2: The individual-identifying sequence in REGEX format ('Seq').

The REGEX format of the first row in the example below reads, 'the entirety of AGGTTCGAGCGAAAATCGA (indicated by brackets) or (indicated by |) the entirety of CCCAAACAGCATTAAACCT (indicated by brackets)'.

Each sequence within brackets comprises the individual-specific barcode (4 bp), the A or T resulting from the ligation step (1 bp), part or all of the gene-specific primer (14 bp here), all in the appropriate order to match the forward or the reverse sequences, the reverse sequences having previously been reverse-complemented.

e.g.

Name,Seq

Pco_NSW_79,(AGGTTCGAGCGAAAATCGA)|(CCCAAACAGCATTAAACCT)

Pco_VIC_80,(AGTATCGAGCGAAAATCGA)|(CCCAAACAGCATTAATACT)

Phe_NSW_82,(CCCATCGAGCGAAAATCGA)|(CCCAAACAGCATTAAGGGT)

...

Now open R and use the following script (comments indicated by ‘#’).

#import both files into R

readnos<-read.csv("Readnos.csv")

ids<-read.csv("IDs.csv")

#set up a file to which the R console output will be #saved (called "Matches.csv")

sink(file="Matches.csv", append=TRUE)

#set up a 'for' loop to detect matches for each of the 64 #individuals in turn.

for(i in c(1:64)){

#create a vector of the row names in the readnos file #that match the individual ID

posmatch<-c(grep(pattern=ids$Seq[i], x=readnos$Seq))

#In the example given above, R will detect a match in #Read 1 to the first sequence listed for the individual #called Phe_NSW_82 (highlighted in yellow).

# create either a vector of the matching row names (which #correspond to the read numbers), or ‘NA’ (if no matches #were found for that individual)

howlong<-ifelse(length(posmatch)>=1, length(posmatch), 1)

posmatch2<-rep(x=ifelse(length(posmatch)>=1, "some", "none"), each=howlong)

posmatch3<-ifelse(posmatch2>1, posmatch, posmatch2)

#return a data frame containing the read no. and #corresponding individual matches (or 'NA')

tempdata<-data.frame(posmatch3, ids$Name[i])

print(tempdata)

#close the 'for' loop

}

#stop writing to the 'Matches.csv' file

sink()

#The resulting ‘Matches.csv’ file will contain three #columns: R row numbers (can be deleted), read no. and #individual name that matched that read no. You will need #to convert this file to a .txt file before uploading #into Galaxy.
